# Magnesium-Phosphoricum and Schussler’s Bio-Chemistry

**Published:** 1890-01

**Authors:** Daniel W. Clausen

**Affiliations:** Lately of Auburn, N. Y.


### MAGNESIUM-PHOSPHORICUM AND SCHUSSLER'S BIO-CHEMISTRY.

Daniel W. Clausen, M. D., Lately of Auburn, N. Y.

Magnesium-phos. is classed as one of the so-called "Tissue remedies" of Schussler, being considered by that physician the remedy for "diseases having their seat in the nerve fibre cells or in the terminal bulbs of the nerves, in the muscles or in the muscular tissue itself," and as the remedy for all spasmodic conditions; but the homœopath, who has long since learned the utter worthlessness of pathological and chemical theories as indications for the selection of curative agents in the treatment of disease, has also learned that bio-chemistry is not the law of therapeutics. Natural law is universal; its application is without a peradventure. Release an apple from your grasp, and it will never ascend to your zenith, but will invariably fall in a direct line toward the centre of the earth, in obedience to the law of gravity. A law that governs the science of treating human infirmities must also of necessity be a natural law. That there *is* a bio-chemic law, which is also a natural law, not a doubt can reasonably be entertained; but the appropriation of the "tissue salts," and their discrepancies in the living organism, belong to the domain of physiology—normal and perverted—rather than to the domain of therapeutics. If Schussler's bio-chemic theory involved a natural law for the treatment of disease, that law would be universally applicable to all cases of sickness; but, by way of example, how many out of fifty cases of epilepsy, or of any other "spasmodic" affection, could we cure with Magnes-phos., which is, according to Schussler, the true "anti-spasmodic" remedy? Moreover, the fact that Schussler's remedies are limited to twelve mineral salts, to the exclusion of thousands of other resources of nature for the treatment of disease, shows the absurdity of his theory as a basis for therapeutics. If Schussler's remedies have effected cures in some cases—and they undoubtedly have—it is not because the bio-chemic theory furnished any correct indications for the selection of them, but because of their homœopathic adaptability. It is for this reason that the homœopath has a perfect right to take advantage of clinical experiences, even those of Schussler himself. The fifty cases above mentioned by way of example, could not all be cured by one remedy of Schussler's, nor perhaps by any other one remedy, but, if curable, they could be cured by the correct application of the one universal law governing medical treatment —*the law of similars.*

Let bio-chemistry have all due credit for the faithful performance of its work in the organism; but let Homœopathy alone have the credit for exciting the reactionary powers of nature against all morbid processes and influences, and thus preparing the way for bio-chemistry to do its work. If this is clearly understood, it will be seen how infinitesimal doses cause deficiencies in the living organism to be supplied, while they themselves contain not sufficient material to meet the deficiency.

I have seen a scrofulous child perform locomotion with perfect ease, run and jump, in a very few months after the exhibition of only two doses of Calc-ostr.$^{6000}$ (Jennichen), the child having previously been totally unable to stand. Surely there was not sufficient lime in those globules to "supply a deficiency" in the patient.

A misunderstanding of this causes the untaught allopath to laugh at us for administering a few globules of Ferrum$^{200}$ in a case of anæmia, while it causes the materialist in Homœopathy to resort to a trituration bordering closely on the crude drug, if he is not mongrel enough to prescribe a "tonic" or crude tincture from the drug store.

We have selected Magnesium-phosphoricum for discussion this evening, to illustrate the only one and universal principle upon which we may determine its adaptability to any given case of sickness; but our time will permit us to offer but *one* striking indication for its use; and the following case is given by way of illustration.

December 5th, 1889, Mrs. J. D., æt. thirty-four; has had facial neuralgia for the last eight or ten days, with "soreness of the front teeth" (upper and lower), pressure of the teeth together being intolerable. The pain began in the cheek bones, and gradually involved the upper and lower jaws on each side; it is aggravated by eating, also by drinking anything either cold or hot, but is *ameliorated by dry warmth externally applied.* It is a constant ache in all parts involved, but every few seconds a severe throbbing occurs from the cheeks to the jaw-teeth, worse on lying down, when there is also a throbbing from the temples to the top of the head, "as if the head would come off." The patient feels as though she would "go crazy" with the pain when lying down or stooping forward; she also has sharp, lancinating pains passing through the decayed teeth (jaw and front). ℞, *Magnes-phos.* 12x (B. & T.), a powder at 1 P. M. In half an hour or less time the pain was so much worse that she thought she should "go wild" with it. Took another dose at 4 P. M., and in half an hour after this second dose the pain was gone—absolutely gone. At supper-time the same evening she could drink hot or cold with impunity, and could eat on the teeth without soreness. I believe the CM potency would not have allowed any aggravation such as followed the first dose of the 12x; but this preparation (12x) was the only one at hand when the patient was seen. December 8th (three days after), no return of the pain. The indication for *Magnes-phos.* in this case was the *relief from externally applied warmth;* and it would seem, from this case, that *dry warmth* is a special indication. Had the pain been an insupportable pain at night, compelling the patient to rise and walk the floor for relief, we should then have had a striking indication for the exhibition of the *carbonate* rather than the phosphate of Magnesium. Magnes-phos. is worthy of a careful study, and is likely to have a great sphere of usefulness in therapeutics. The last number of the *Medical Advance* contains an elaborate proving of the drug, besides one or more fragmentary provings of the same. The seventh volume of Hering's *Guiding Symptoms* also furnishes characteristics of Magnes-phos.; but we fear that too many "additions from current literature" have been made to the symptoms given in this volume, to merit our confidence in them all as "guiding symptoms." A very few more words, and I shall have finished. Magnes-phos. does not require to be given in "hot water," as Schussler advises. If the drug is homœopathically indicated, it may be administered in all faith, from the 30th to the CM, dry on the tongue, or dissolved in cold water.

